# Unicuspid unicommissural aortic valve

**DOI:** 10.34172/jcvtr.33170

**Published:** 2024-09-20

**Authors:** Nataraju Komalamma Girish, Niraj Nirmal Pandey, Priya Jagia, Sandeep Singh

**Affiliations:** ^1^Department of Cardiovascular Radiology & Endovascular Interventions, All India Institute of Medical Sciences, New Delhi-110029, India; ^2^Department of Cardiology, All India Institute of Medical Sciences, New Delhi-110029, India

**Keywords:** Aortic valve, Computed tomography angiography, Cardiac-gated imaging techniques

## Abstract

We describe a case of a 30-year-old woman with gradually increasing dyspnoea on exertion where CT angiography revealed a unicuspid unicommissural morphology of the aortic valve. The present report highlights the anatomical and embryological aspects of this rare anatomical variant as well as the associated cardiovascular abnormalities.

## Description of the case

**Figure 1:**
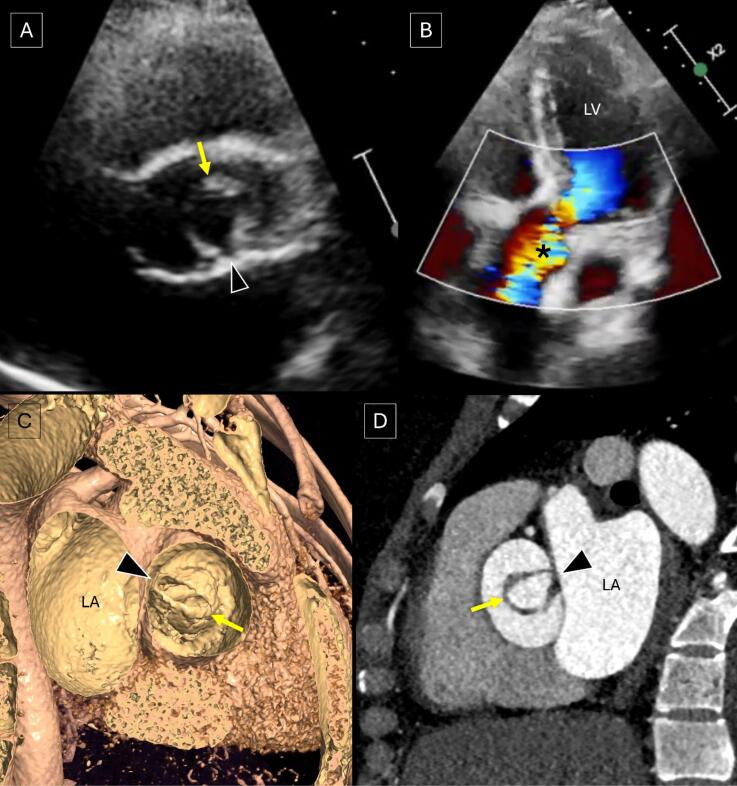
Transthoracic echocardiography image of the aortic valve in the short axis plane **(A)** depicts the aortic valve with a single commissure (arrowhead), a rounded, leaflet-free edge (yellow arrow) opposite to the commissure and eccentric valvular opening during systole. Doppler image in the apical four chamber view **(B)** reveals eccentric turbulent flow (*) across the aortic valve suggesting aortic stenosis. Virtual dissection image **(C)** of CT angiography profiling the aortic valve from the ascending aortic side (bird’s eye view) and oblique axial image **(D)** across the aortic valve demonstrate a unicuspid aortic valve with a single commissure (arrowhead) and an opposite rounded, leaflet-free edge (yellow arrow). [LA: left atrium; LV: left ventricle]

 A 30-year-old female presented with gradually increasing dyspnoea on exertion (NYHA II). She had a previous history of undergoing stenting for coarctation of the aorta 1 year back. Transthoracic echocardiography demonstrated the aortic valve having a single commissure, a rounded, leaflet-free edge opposite to the commissure and eccentric valvular opening during systole with severe aortic stenosis (peak gradient of 71 mmHg and mean gradient of 41 mmHg) and normal left ventricular systolic function (Figure 1A-B, Supplementary file 1, video 1 and Supplementary file 2, video 2). A cardiac CT angiography was performed which revealed the aortic valve to be a unicuspid unicommissural valve (Figure 1C-D, Supplementary file 3, video 3). The ascending aorta was dilated (measuring ~ 41 mm in diameter) and the stent across the segment of coarctation was patent (Supplementary file 4, figure S1). Both the coronary arteries were seen arising from a single coronary sinus with adjacent ostia.

## Discussion

 Unicuspid aortic valve is a rare congenital occurrence, with a prevalence of 0.02% in the adult population.^1^ It has been reported in approximately 0.019% patients undergoing transthoracic echocardiography and in approximately 5.59% patients undergoing aortic valve surgery.^2^ Unicuspid aortic valve can be further classified into two types depending upon the presence (unicuspid unicommissural) or absence (unicuspid acommissural) of the lateral attachment of the cusps to the aorta orifice. A unicuspid aortic valve is postulated to develop secondary to the failure of separation of the 3 aortic cusps before birth which in turn develop from the embryonic tubercules of the aortic trunk. Isolated aortic stenosis is the most common valvular abnormality associated with unicuspid aortic valve. Other associated cardiovascular abnormalities include aortic valvular regurgitation, aortic aneurysm/ dissection/ coarctation, and the presence of a patent arterial duct.^1^

## Conclusion

 Unicuspid aortic valve is a rare variant and, based on the presence or absence of the lateral attachment of the cusps to the aorta orifice, can be unicommissural or acommissural respectively. While commonly presenting with isolated aortic valvular stenosis, it can also be associated with aortic valvular regurgitation, aortic aneurysm/ dissection/ coarctation, and a patent arterial duct. The present report highlights the anatomical and embryological aspects of this rare anatomical variant as well as the associated cardiovascular abnormalities.

## Competing Interests

 The author(s) declared no potential conflicts of interest with respect to the research, authorship, and/or publication of this article.

## Ethical Approval

 All procedures performed in studies involving human participants were in accordance with the 1964 Helsinki declaration and its later amendments or comparable ethical standards. Written informed consent was obtained from the patient for the publication of this case report and accompanying images.

## Supplementary Files


Supplementary file 1 contains.Transthoracic echocardiography cine loop of the aortic valve in the short axis plane depicting the eccentric opening during systole with a single commissural attachment and a rounded, leaflet-free edge opposite to the commissure.

Supplementary file 2 contains Transthoracic echocardiography Doppler imaging in the apical four chamber view reveals eccentric turbulent flow across the unicuspid aortic valve with normal left ventricular systolic function.

Supplementary file 3 contains Cine visualization of CT angiography in a plane passing across the aortic valve leaflet reveals a unicuspid aortic valve with a single commissure.

Supplementary file 4 contains figure S1.
