# Supplementary material for: Unicuspid unicommissural aortic valve
Source: J Cardiovasc Thorac Res. 2024 Sep 20;16(3):198–9. doi: 10.34172/jcvtr.33170 (PMC11489640; doi:10.34172/jcvtr.33170)
Supplement: Supplementary file 4 — contains figure S1. [file jcvtr-16-198-s004.pdf]

J Cardiovasc Thorac Res, 2024, 16(3), 198-S4
doi: 10.34172/jcvtr.33170
http://jcvtr.tbzmed.ac.ir

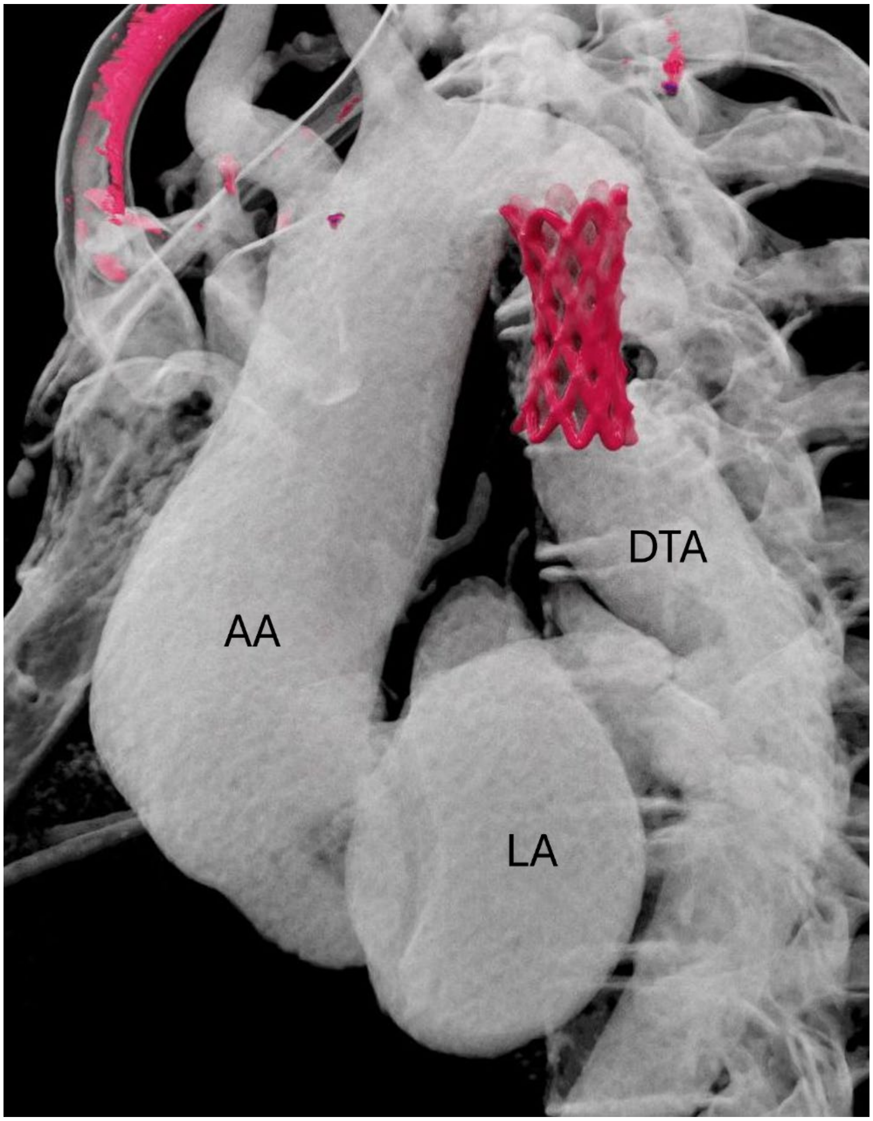

Supplementary figure 1: Volume rendered image reveals mild ascending aortic (AA) dilatation with a patent stent in the descending thoracic aorta (DTA) across the segment of coarctation. [LA: left atrium]
